# The Impact Hazard Assessment for Near-Earth Asteroid 2024 YR_4_

**DOI:** 10.1007/s40295-026-00576-0

**Published:** 2026-03-05

**Authors:** Davide Farnocchia, Marco Fenucci, Fabrizio Bernardi, Lisa Bedini, Alessia Bertolucci, Steven R. Chesley, Paul W. Chodas, Maxime Devogèle, Laura Faggioli, Francesco Gianotto, Marco Micheli, Ryan S. Park, Giovanni B. Valsecchi

**Affiliations:** 1 Jet Propulsion Laboratory, California Institute of Technology, 4800 Oak Grove Dr., Pasadena, CA 91109 USA; 2 NEO Coordination Centre, European Space Agency, Largo Galileo Galilei 1, 00044 Frascati, RM Italy; 3 SpaceDys s.r.l., Via Mario Giuntini 63, Navacchio di Cascina, 56023 PI Italy; 4 IAPS, INAF, Via Fosso del Cavaliere 100, 00133 Rome, RM Italy

**Keywords:** Asteroids, Asteroid dynamics, Near-Earth objects, Astrometry

## Abstract

Discovered by the ATLAS survey on December 27, 2024, asteroid 2024 YR_4_ represented one of the most significant impact risks ever detected. Roughly 60 m in size, this asteroid reached a 3% probability of impacting the Earth on December 22, 2032. We discuss the orbit and impact analyses carried by the three impact monitoring systems Sentry, Aegis, and NEODyS. The orbit solution proved to be remarkably stable and the impact probability behaved according to expectations as the uncertainty decreased thanks to additional observations, i.e., the impact probability initially increased as the Earth remained within the core of the uncertainty region and then dropped as the Earth moved towards the tail and eventually outside of the uncertainty region. Although the risk of an Earth impact has been ruled out, there remains a 4% probability of a lunar impact on December 22, 2032. To confirm or more likely rule out that possibility, we need to wait until new astrometric data become available, e.g., during the 2028 apparition.

**Supplementary Information:**

The online version contains supplementary material available at 10.1007/s40295-026-00576-0.

## Introduction

While asteroids of a few meters in size can reach the Earth on a yearly basis [1–5],^1^ impacts with asteroids capable of significant damage to the ground are far rarer [6]. Three impact monitoring systems constantly assess the risk of asteroid impacts for the entire catalog of near-Earth objects by estimating the probability of an Earth impact over the next 100 years: Sentry, developed, maintained, and operated by the Center for Near-Earth Object Studies (CNEOS) at NASA’s Jet Propulsion Laboratory,^2^ Aegis at the ESA NEO Coordination Center (NEOCC),^3^ and NEODyS, which was developed at the University of Pisa and is operated by SpaceDyS^4^ [7–9]. Among the three systems there are several differences in the algorithms used (e.g., Sentry uses an impact-observation method [8] while Aegis and NEODyS are based on the Line-of-Variations [10]), the software packages, and the statistical treatment of the observational data, e.g., data weights and outlier rejections. These differences and the comparison of the results allow an assessment of the sensitivity to the different model assumptions and configurations, and in turn provide increased robustness to the overall impact monitoring system.

The asteroid impact risk is typically ranked using two scales: the Torino Scale [11], which is an integer number from 0 to 10 based on the probability of impact and the impact energy, and the Palermo Scale [12], which measures the risk from a specific potential asteroid impact relative to the background risk from the entire population at similar sizes. All cataloged asteroids currently rank 0 on the Torino Scale for the next 100 years, meaning that the probability of collision is too small to be of significance or that the object is too small to cause any material damage. Since the advent of asteroid impact monitoring, (99942) Apophis and its possible collision in 2029 represented the highest risk ever reported.^5^ In December 2004, the impact probability reached a peak of 2.7% and Apophis ranked 4 on the Torino Scale and +1 on the Palermo Scale, before precovery observations from March 2004 reported by Spacewatch ruled out the possible impact [13].^6^ As of the end of 2024, about 60 objects reached a Torino Scale of 1 and only (144898) 2004 VD17 in February 2006 reached a Torino Scale of 2, but all of them eventually dropped back to 0 as additional observations were collected.^7^

A recent addition to the category of notable cases is asteroid 2024 YR_4_, which in early 2025 reached a Torino Scale of 3 for an impact in December 2032. The impact probability reached a peak of 3%, which represented a record for the size of 2024 YR_4_ (approximately 40–90 m). In this paper, we provide an account of the orbit determination and impact hazard assessment analyses performed since the discovery of 2024 YR_4_ through the end of the discovery apparition, which ultimately led to ruling out any Earth impact risk.

## Discovery and First Impact Analysis

After making a close approach to Earth at a distance of 830,000 km on December 25, 2024, asteroid 2024 YR_4_ was discovered on December 27, 2024, by the ATLAS survey [14] at an *o*-band apparatent magnitude of 16.5 (MPEC 2024-Y140).^8^ The asteroid was designated by the Minor Planet Center [15] based on 17 observations from five observatories over 31 hours, including prediscovery observations from the Catalina Sky Survey [16]. From photometric measurements, the absolute magnitude of 2024 YR_4_ was estimated to be $H\sim 24$, which can be converted to diameter [17] for a given geometric albedo $p_V$:

$$ D = 1329\text { km}\frac{10^{-H/5}}{\sqrt{p_V}}\ . $$

For $p_V$ between 25 and 5%, the likely diameter range is between 40 and 90 m.

**Fig. 1:**
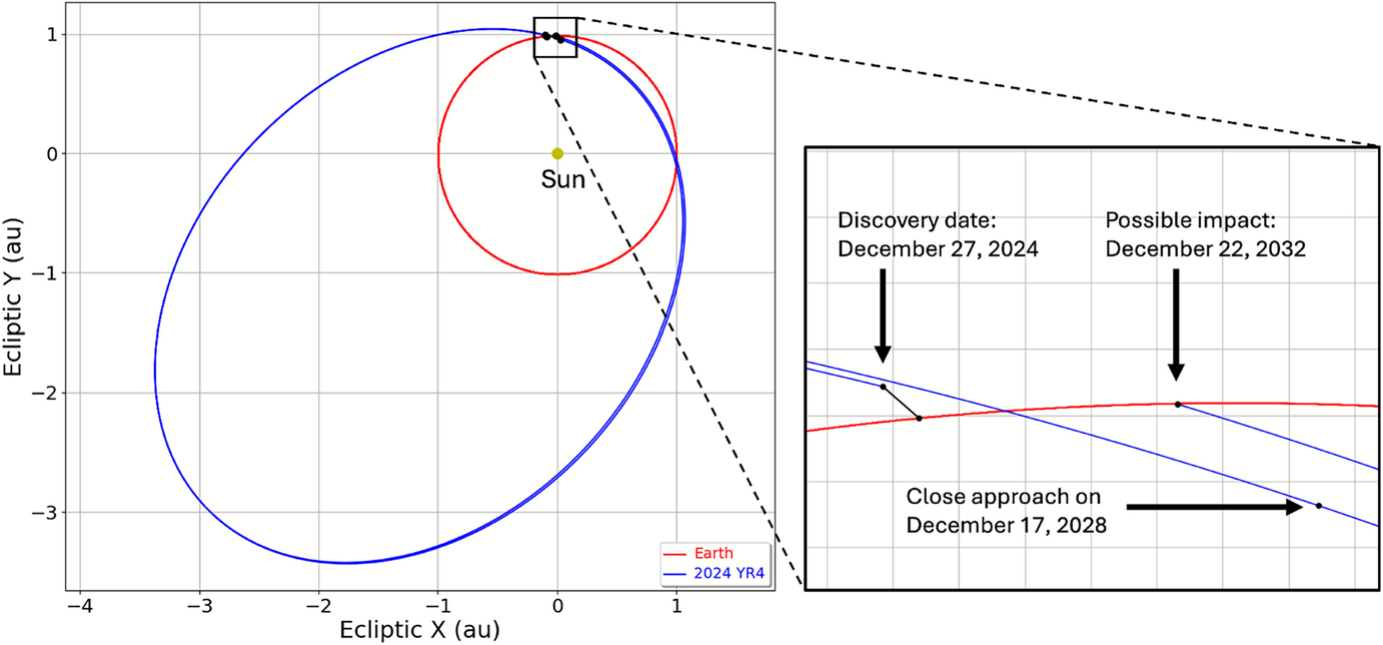
Orbit of 2024 YR_4_ (blue) compared to that of the Earth (red) projected on the ecliptic plane

**Fig. 2:**
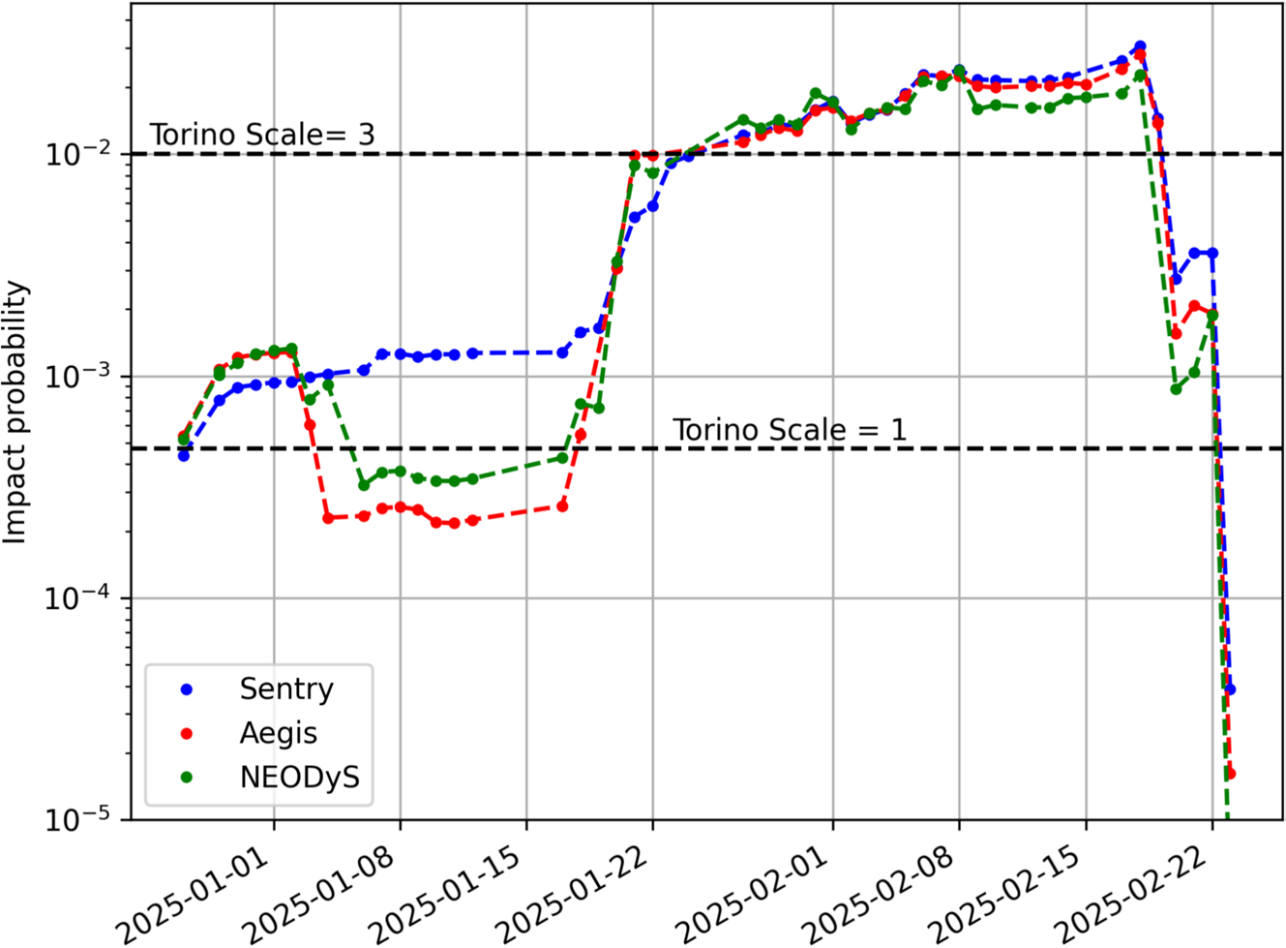
Probability of an Earth impact on December 22, 2032, for 2024 YR_4_ as a function of time for the three impact monitoring systems: Sentry, Aegis, and NEODyS. The black dashed lines represent the impact probability thresholds for reaching Torino Scale levels 1 (from 0) and 3 (from 1) given the size of 2024 YR_4_

As shown in Fig. 1, the orbit of 2024 YR_4_ after the close approach on December 25, 2024, is characterized by an eccentricity of 0.66, a semimajor axis of 2.5 au, and an inclination of 3.4 deg. The orbital period is 4.0 years, which puts 2024 YR_4_ into a 4:1 mean motion resonance with the Earth and brings it back into the Earth’s neighborhood every four years. While the 2028 close approach is at a safe distance of 0.05 au, based on the observational data from the discovery MPEC, Sentry, Aegis, and NEODyS all identified the possibility of an Earth impact on December 22, 2032. The resulting Palermo Scale was greater than $-2$, i.e., the threshold corresponding to 1% of the background risk and used to trigger technical verification. Therefore, the results of the different systems were compared finding substantial agreement. Sentry reported an impact probability of $IP_S = 4.4\times 10^{-4}$, Aegis $IP_A = 5.4\times 10^{-4}$, and NEODyS $IP_N = 5.2\times 10^{-4}$. As shown by the leftmost points in Fig. 2, for the size of 2024 YR_4_, these initial impact probability values are close to the boundary between Torino Scales 0 and 1, which was crossed by Aegis and NEODyS but not yet by Sentry. The impact velocity would be 17.3 km/s.

## Impact Probability Evolution

During the discovery apparition, 2024 YR_4_ was tracked with a total of 504 observations reported from 63 different observatories. 2024 YR_4_ was discovered after a close approach and on an outbound trajectory, which brought it farther away and made it fainter with time. Therefore, the number of observations and of telescopes capable of observing 2024 YR_4_ decreased with time, as shown in Table 1. The last reported observation was from the James Webb Space Telescope (JWST) on May 11, 2025 (MPEC 2025-L19).^9^ The full dataset is available from the Minor Planet Center.^10^^,^^11^ A companion paper part of this issue discusses the astrometric measurement process for many of the key observations collected during the discovery apparition of 2024 YR_4_ [18].

**Table 1:** Number of observations, unique observatories that observed 2024 YR_4_, and final 2024 YR_4_’s V-band magnitude for different lunations

Time period	Number of observations	Number of stations	Final V-mag
2024-12-25 – 2025-01-13	225	40	21
2025-01-13 – 2025-02-12	184	23	24
2025-02-12 – 2025-03-14	91	15	26
2025-03-14 – 2025-04-13	3	2	27
2025-04-13 – 2025-05-12	1	1	28
Total	504	63	

During this apparition, we frequently refined the orbit estimate and probability of an impact in 2032 as soon as new observations became available, as discussed in the subsections below. It is worth pointing out that until January 20, 2025, there were two separate virtual impactors (VI), i.e., separate subsets of the orbital uncertainty leading to an impact [19], for 2032. While it was initially unclear which one would be more significant, here we focus on the one that eventually reached an impact probability greater than 1%. Since discovery, there have also been VIs at later epochs with a much lower impact probability. In fact, at the end of the apparition, we are left with a possible impact in 2047 with a very small probability of less than $10^{-5}$ and a Palermo Scale of $-4.23$.^12^^,^^13^^,^^14^ For context, as of December 10, 2025, the Sentry risk list includes 665 objects with an impact probability larger than $10^{-5}$ and 65 objects with a Palermo Scale greater that 2024 YR_4_’s.

### First Phase Through January 22, 2025

The initial phase until January 22, 2025, was characterized by routine coordination between the teams operating Sentry, Aegis, and NEODyS. During this time, the Palermo Scale was always $>-2$ and therefore the publication of the results only happened after confirming that each system found the possible impact and with similar impact probabilities. As usual for the three teams, the verification happened by exchanging results via email.

As shown by Fig. 2, new observations made available on December 29, 2024 (MPEC 2024-Y189),^15^ led to an increase in impact probability and all three systems now ranked 2024 YR_4_ as Torino Scale level 1. This upward trend was consistent for weeks in the Sentry results. Aegis and NEODyS produced results close to each other, but with a different behavior than the Sentry ones. Their impact probability initially increased and then decreased enough to lower the Torino Scale level to 0.^16^ This early January dip represents the most significant difference in impact probability between the three systems.

A difference in impact probability is not necessarily an issue and could be due to several reasons, the most important one being different assumptions in data weights. The automatic orbit solutions used by Sentry are based on a scheme derived from a statistical analysis of the performance of the different observatories [20]. Moreover, based on uncertainty information provided by observers in the IAU ADES format^17^ and inspection of the observation residuals, weights and outlier rejections can manually be selected by the orbit determination analyst for the more significant objects as was the case for 2024 YR_4_. The Aegis solution was computed based on the same general weighing scheme [20], though tighter uncertainties were assumed for astrometric observations reduced against the Gaia star catalogs [21–23] and manual weights were set for observations reported by NEOCC observers. Another difference is that Sentry accounts for an observation time uncertainty [24], set to 1 s for all observations of 2024 YR_4_, while Aegis and NEODyS did not include a time uncertainty during this first phase.

**Fig. 3:**
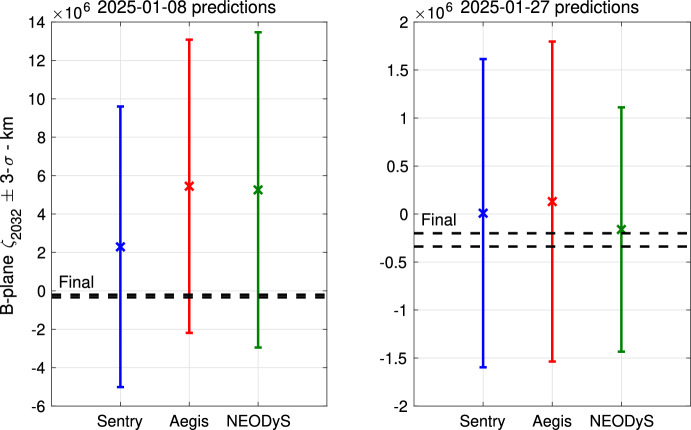
Comparison of the 2032 B-plane’s $\zeta _{2032}$ prediction for Sentry, Aegis, and NEODyS. The left panel corresponds to the orbital solutions computed on January 8, 2025, while the right panel corresponds to the orbital solutions computed on January 27, 2025. The black lines encompass the 3-$\sigma$ range for $\zeta _{2032}$ as computed at the end of the apparition

As an example, we can take a closer look at the three different analyses performed on January 8, 2025, when the impact probabilities differed by a factor of 5: $IP_S = 1.3 \times 10^{-3}$, $IP_A = 2.6 \times 10^{-4}$, and $IP_N = 3.7 \times 10^{-4}$. However, the left panel of Fig. 3 shows how the three predictions for the $\zeta$ coordinate on the 2032 B-plane [25, 26] are statistically consistent with each other and with the final estimate at the end of the apparition. At this time, the impact solution is 1-$\sigma$ from the nominal solution for Sentry, while it is closer to a 2-$\sigma$ offset for Aegis and NEODyS.

Around mid-January, the Aegis and NEODyS probabilities reversed course and increased sharply, while the Sentry probability kept increasing monotonically.

### Reaching 1%

On January 22, 2025, the impact probabilities computed by the three systems were $IP_S = 0.58\%$, $IP_A = 0.99\%$, and $IP_N = 0.82\%$. Therefore, it became clear that there was a significant possibility that the impact probability could cross 1%, a significant threshold that would result in a Torino Scale level of 3 and trigger formal notification by the International Asteroid Warning Network (IAWN) [27].^18^

**Fig. 4:**
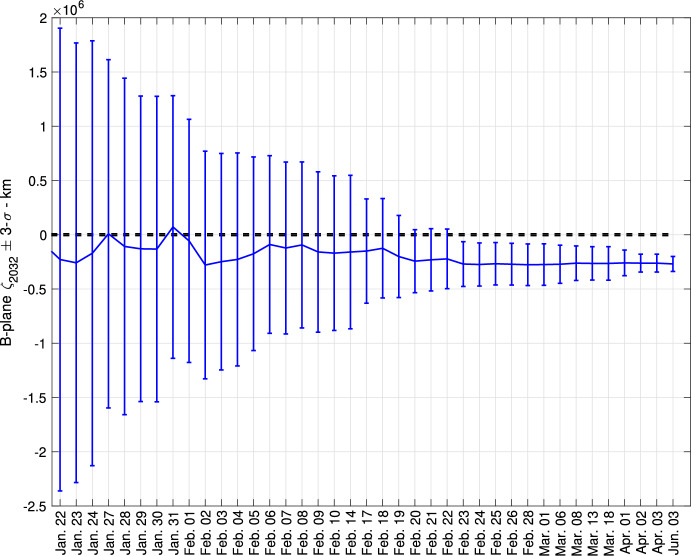
Evolution of the Sentry $\zeta _{2032}$ prediction as function of the date of the orbit solution. The error bars correspond to the 3-$\sigma$ level. The dashed, black lines encompass the values of $\zeta _{2032}$ leading to an impact

As shown by Fig. 4, the $\zeta _{2032}$ prediction on that date was close to $\zeta _{2032} = 0$, which corresponds to an impact; the impact solution was only 0.3-$\sigma$ from the Sentry nominal. As a result, the most likely outcome of additional observations was a decrease in uncertainty and, in turn, an increase in impact probability unless the nominal prediction moved farther away from the Earth. By using the approximation of a 1-D uncertainty described by the $\zeta$ direction on the B-plane and a normal distribution with mean $\mu _\zeta$ and standard deviation $\sigma _\zeta$, the impact probability *IP* can be computed as:

1 $$\begin{aligned} IP \sim f(0) c \ \ ,\ \ f(\zeta ) = \frac{1}{\sqrt{2\pi }\sigma _\zeta } e^{-(\zeta - \mu _\zeta )^2/2\sigma _\zeta ^2} \end{aligned}$$

where *c* is the chord corresponding to the intersection of the Line of Variations and the impact cross section on the B-plane. As new observational data becomes available, $\mu _\zeta$ and $\sigma _\zeta$ will change, and the first order correction in impact probability can be assessed using the partial derivatives:

2 $$\begin{aligned} \frac{\partial IP}{\partial \mu _\zeta } = -\frac{\mu _\zeta IP}{\sigma _\zeta ^2}\ \ ,\ \ \frac{\partial IP}{\partial \sigma _\zeta } = \frac{IP}{\sigma _\zeta }\left( \frac{\mu _\zeta ^2}{\sigma _\zeta ^2} - 1\right) \end{aligned}$$

A $\mu _\zeta$ more distant from zero always decreases the probability. On the other hand, a decreasing $\sigma _\zeta$ leads to an increased probability if the impact is within 1-$\sigma$ (i.e., $|\mu _\zeta | < \sigma _\zeta$), and a decreased probability if the impact is more than 1-$\sigma$ away.

On January 22, 2025, the Sentry, Aegis, and NEODyS teams met virtually to carefully review the situation and compare results. The goal of this coordination effort was not to artificially arrive at the same results, but rather understand the reasons for the different results and whether the assumptions made by each team were justifiable:

- We reviewed observations where the automatic weighting schemes differed, assessing whether the different data weights were reasonable and how much those differences affected the orbit solution.
- We tested the contribution of each observation tracklet to assess the sensitivity of the orbital solution on it. For the observations that had the most leverage, we contacted observers to request detailed feedback on their data to better decide how to weight their observations in a realistic but still conservative way.
- Some observation batches showed possible biases, in which case we also contacted the corresponding observers to know if there was any possible problem with that observation run (e.g., time biases, winds, poor seeing). Based on the reply, we assessed the possibility of deweighting or even rejecting such data.
- We tested different assumptions on the timing uncertainties, especially for the earlier observations in the dataset when 2024 YR_4_ had a higher plane-of-sky rate of motion. Aegis introduced timing errors, using the observer reported information when available and assuming a 1 s uncertainty otherwise.
- We remeasured astrometry from ATLAS and Catalina.
- For all systems, the force model was based on JPL’s Planetary Ephemeris DE441 [28], though some differences were present the selection of perturbing asteroids [29] and relativistic formulation [30, 31]. As expected, we found that any difference in the force model was dwarfed by the effect of the different statistical treatment of the astrometry.

The three teams kept meeting the following days and weeks, initially on a daily cadence upon the publication of updated observation datasets by the Minor Planet Center. As the situation stabilized and the volume of reported observations decreased, the frequency of the meetings was adjusted based on actual need. After each meeting, the three groups independently revised their setup and the different orbit solutions were compared. The results were published upon anonymous consensus that they were consistent and reliable.

To demonstrate the benefits of this coordination and review effort, the right panel of Fig. 3 shows how closer the three different predictions $\zeta _{2032}$ are when computed on January 27, 2025. Moreover, the impact probability evolutions (Fig. 2) became much closer. Finally, Fig. 4 shows the Sentry $\zeta _{2032}$ prediction evolution starting from January 22, 2025; the orbit solution is remarkably stable over time.^19^

**Fig. 5:**
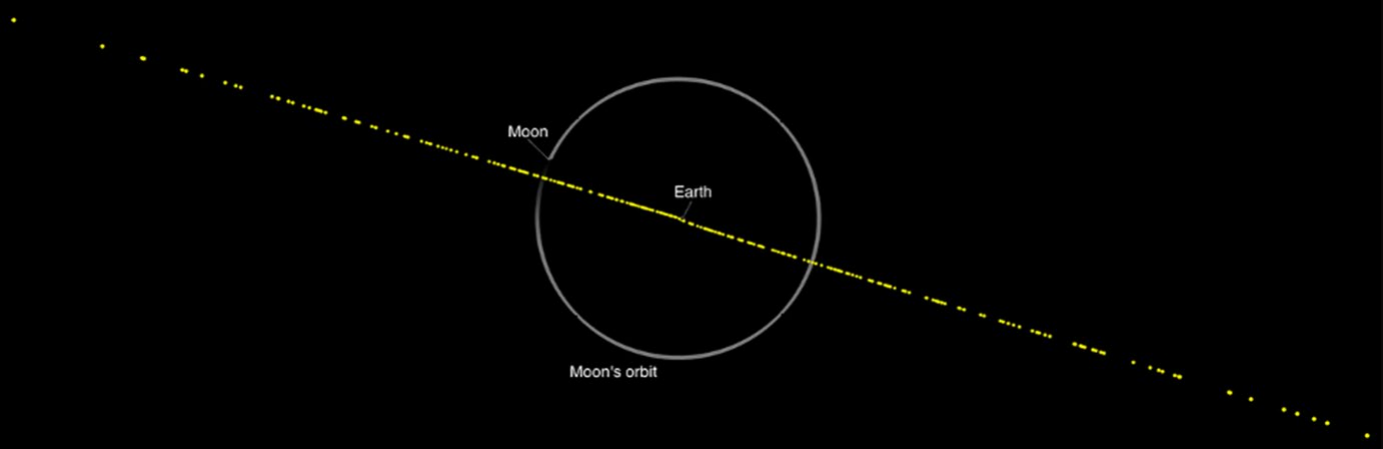
Position uncertainty of 2024 YR_4_ at the time of the December 2032 close approach to Earth. The yellow dots are 300 Monte Carlo samples drawn from JPL solution 35, which was computed on January 27, 2025, when the Sentry impact probability was 1.2%. The positions of the Earth and the Moon are labeled, and the orbit of the Moon is shown for reference

**Fig. 6:**
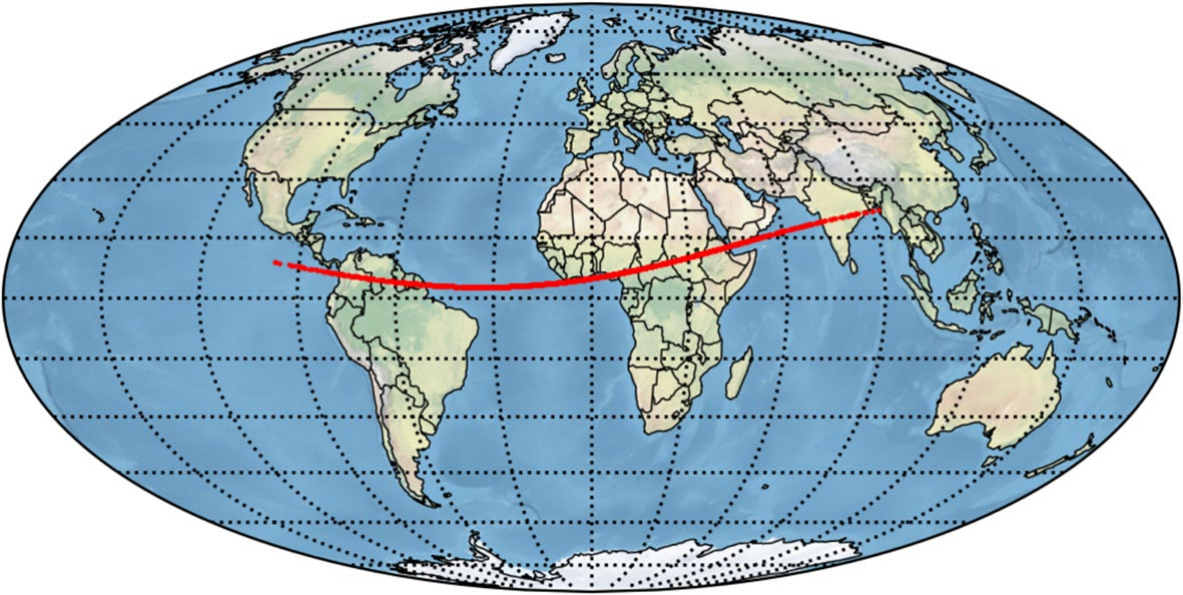
Impact corridor based on 1000 randomly drawn impacting samples for an altitude of 100 km above the WGS-84 reference ellipsoid. The impact times range from 13:52 (west) to 14:16 UTC (east) on December 22, 2032. Thanks to additional data, the possible impact has entirely been ruled out

On January 27, 2025, for the first time all three systems estimated an impact probability greater than 1%: $IP_S = 1.2\%$, $IP_A = 1.1\%$, and $IP_N = 1.4\%$. On January 29, 2025, with the results substantially unchanged, IAWN issued their first ever formal notification of a possible asteroid impact to the Space Mission Planning Advisory Group (SMPAG) and the United Nations Office of Outer Space Affairs (UNOOSA).^20^ These IAWN notifications are issued for asteroids larger than 10 m with a probability greater than 1% of an impact within the next 50 years. At this point, the size of 2024 YR_4_ was uncertain and estimated from photometry to be between 40 and 90 m. JWST observations in March 2025 later constrained the diameter to 60 ± 7 m [32]. Figure 5 shows the position uncertainty of 2024 YR_4_ relative to Earth at the time of the close approach on December 22, 2032, as computed on January 27, 2025. Despite the large uncertainty and the small impact probability, the possible impact locations can be computed in the unlikely case 2024 YR_4_ was on an impact trajectory. Figure 6 shows the impact corridor based on 1000 randomly sampled impact trajectories.

### Impact Probability Peak and Fall

**Fig. 7:**
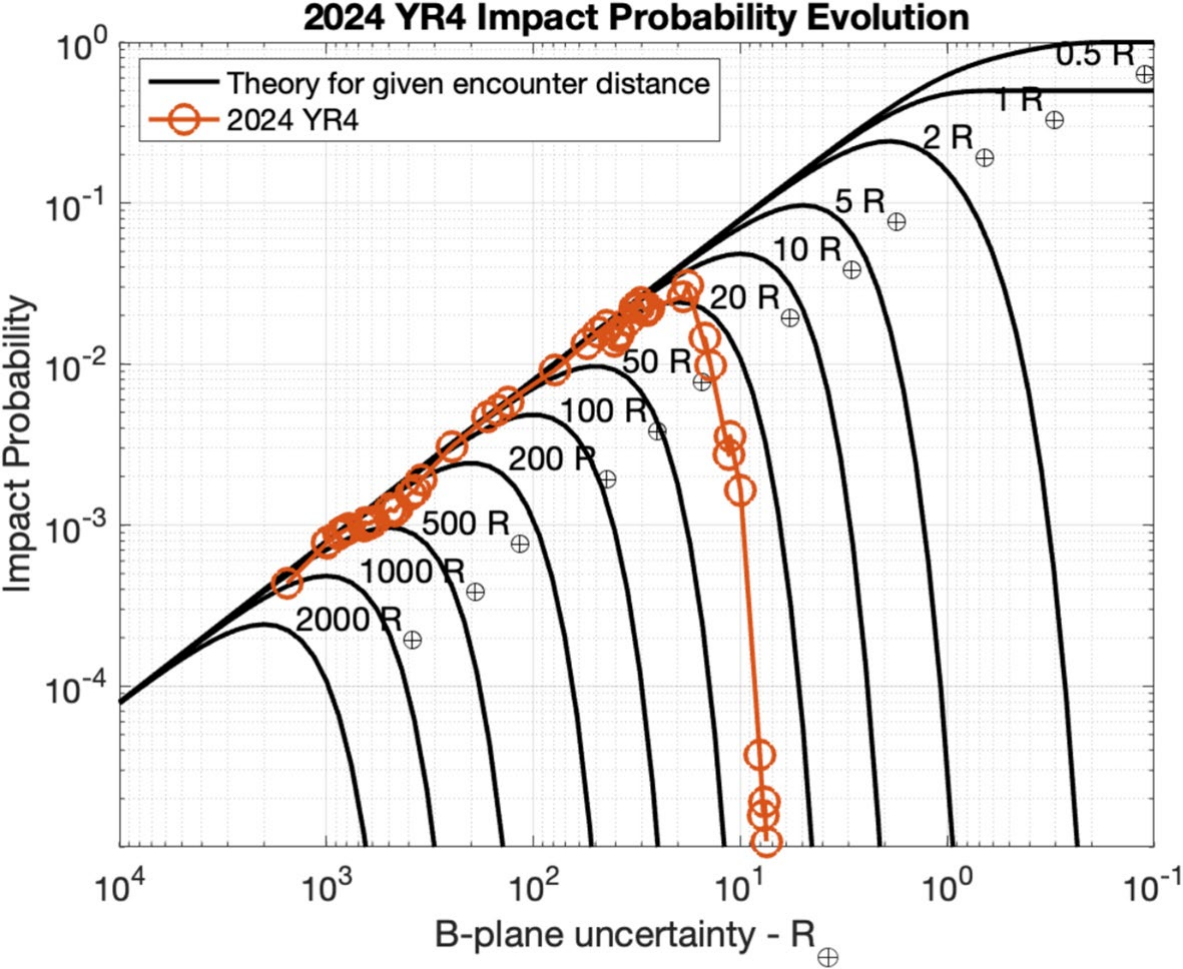
Impact probability curves for different fixed miss distances (in Earth radii $R_\oplus$) as a function of the decreasing 1-$\sigma$ uncertainty of $\zeta _{2032}$. The circles represent the probabilities as computed by Sentry, which should roughly follow one of the black curves, depending on what the true encounter distance turns out to be. However, the match is always going to be imperfect given the unavoidable effects of observational noise

While it is not possible to predict the inherently stochastic evolution of the impact probability, the general trend is for the probability to increase, reach a peak, and then decrease. In the unlikely case that the asteroid were in fact on an impact trajectory, then the probability would keep increasing until 100%. This general behavior is shown in Fig. 7 where each curve shows the impact probability evolution for a fixed miss distance as a function of the decreasing uncertainty on the B-plane.

**Fig. 8:**
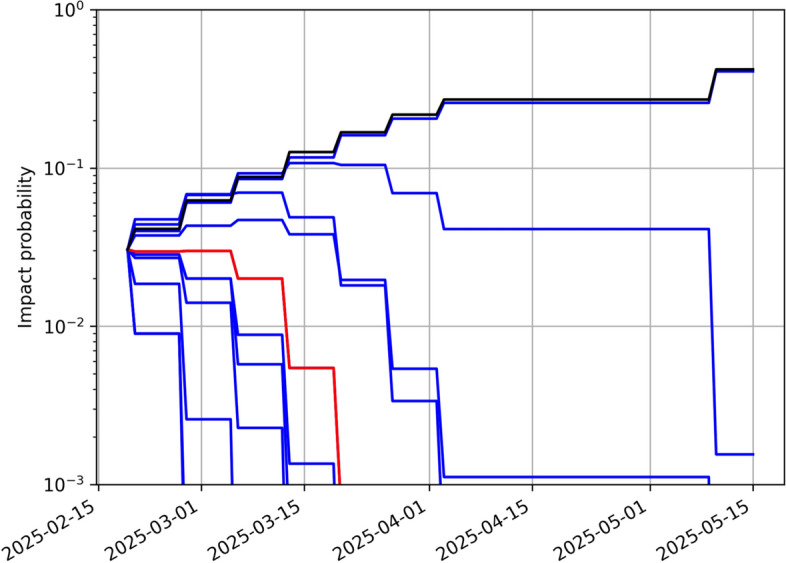
Ten possible impact probability evolutions after February 18, 2025. Each evolution tracks a random sample within the uncertainty region assuming future observations from the ground until early April and from JWST until May. The red curve corresponds to the nominal solution and the black curve to the worst case scenario

At the time the impact probability crossed 1%, the Sentry nominal close approach geocentric distance was only 6900 km, and the impact 0.001-$\sigma$ away. Therefore, an increase in impact probability was to be expected as new data shrank the uncertainty. Figure 2 confirms this expectation with the impact probability that kept increasing until February 18, 2025, when it reached $IP_S = 3.1\%$ ($IP_A = 2.8\%$ and $IP_N = 2.3\%$). At this point, we are closer to the 1-$\sigma$ threshold (Fig. 4) and the impact probability should soon start decreasing if the uncertainty shrinks and the nominal prediction does not move closer to the impact. To assess this possibility, Fig. 8 shows the future probability evolution for 10 orbit samples randomly drawn from the orbit covariance. For seven of them (including the nominal), the probability drops well below 1% with future ground-based observations through early April. For one sample, the impact probability only drops below 1% with JWST astrometry in May. Finally, for two of them the probability increases above 10%, implying that the uncertainty region converges on the Earth as it shrinks over time.

With the data reported on February 19, 2025, the $\zeta _{2032}$ uncertainty decreases from 153,000 to 126,000 km, and the nominal miss distance increases from 120,000 to 200,000 km. As a result, the impact is now 1.5-$\sigma$ away and the impact probability drops to $IP_S = 1.5\%$ ($IP_A = 1.4\%$ and $IP_N = 0.7\%$).

The downward trend is confirmed in the following days: on February 20, 2025, all three systems estimate an impact probability $<1\%$ and the Torino Scale level drops to 1, and on February 23, 2025, the probability drops below $10^{-4}$, at which point the Torino Scale was back to 0. On February 24, 2025, IAWN issued a new notification to formally report the drop in impact probability.^21^ Figure 4 shows how the impact eventually falls well outside the 3-$\sigma$ range for $\zeta _{2032}$, thus ruling out the impact.

## Possible Impact on the Moon

While the data collected during the discovery apparition ruled out the possible Earth impact for 2024 YR_4_, there still remains the possibility of a collision on the Moon. In fact, as the B-plane uncertainty shrank away from the Earth, the uncertainty region converged towards the Moon.

**Fig. 9:**
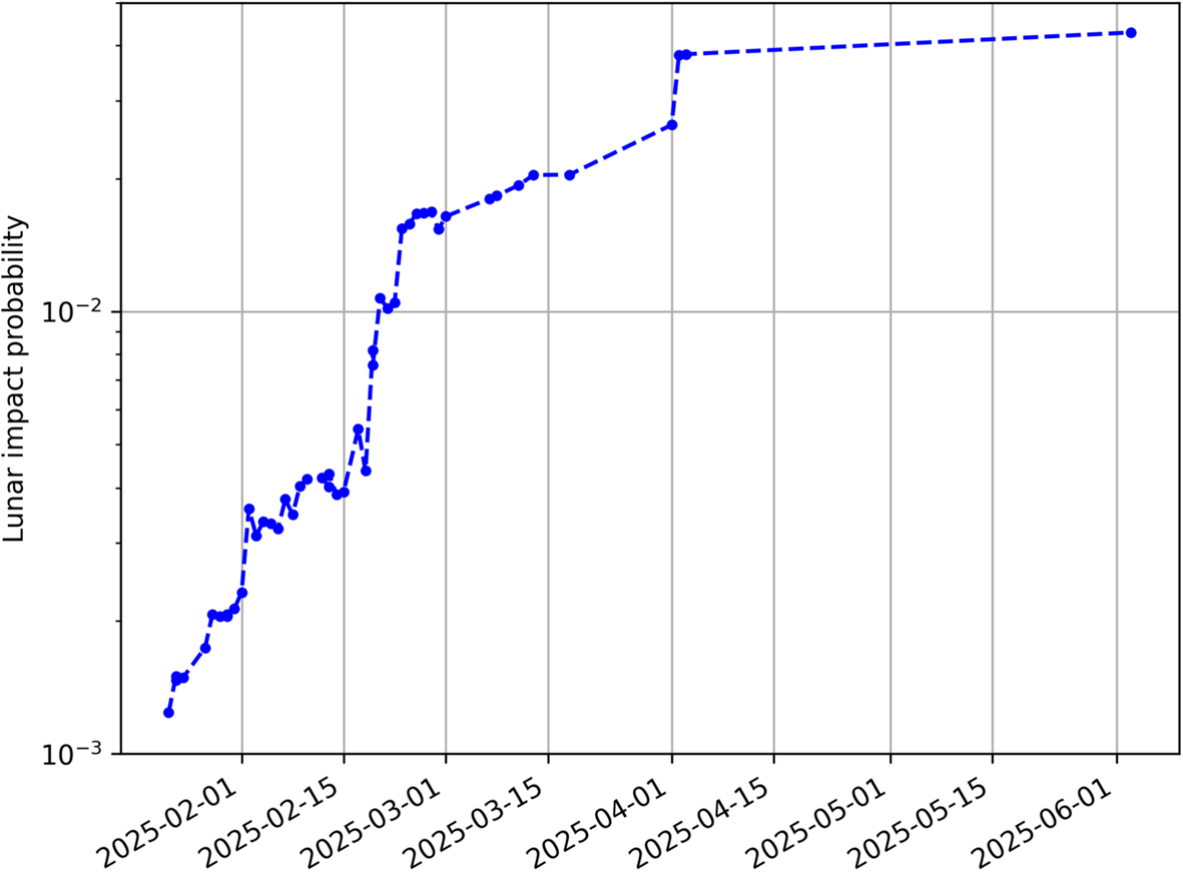
Evolution of the probability of a lunar impact as a function of the date of the orbit solution, starting on January 22, 2025, to the end of the apparition

While Sentry, Aegis, and NEODyS only analyze the possibility of Earth impacts, a linearized approximation of the impact probability can be computed by mapping the covariance onto the Moon’s B-plane [26]. On January 22, 2025, the linearized impact probability for the Moon was greater than 0.1%. For a more reliable impact probability estimate, we resorted to a Monte Carlo simulation with 100,000 samples. Figure 9 shows the evolution of the impact probability for the Moon as a function of time, which corresponds to the date of the corresponding orbital solution. The main trend is an increasing impact probability, though some fluctuations are present due to statistical variations of the orbit solution and Monte Carlo noise.

With the complete dataset from the discovery apparition, the nominal selenocentric closest approach is at 3000 km and the (lunar) B-plane uncertainty is 25,000 km. The resulting impact probability is 4.3% as estimated using a Monte Carlo simulation. The impact velocity would be 14.1 km/s. A more conclusive assessment needs more observations, which will be attempted in early 2026 by JWST [33] and are possible from the ground in 2028.

## Discussion

2024 YR_4_ represented the most significant asteroid impact hazard to Earth since Apophis in 2004 [13]. On January 27, 2025, the impact probability crossed 1% and triggered the first ever IAWN notification. On February 18, 2025, the impact probability reached a peak of 3.1%, which is the highest recorded for any object larger than about 10 m. In the following days, the probability dropped quickly. As shown in Fig. 7, this is the typical behavior: for a given miss distance, the probability initially increases, then reaches a peak, and eventually drops abruptly. The smaller the miss distance, the higher is the peak in impact probability and the tighter the requirements on the prediction uncertainty to rule out the impact. Since the miss distance is not known ahead of time, one cannot predict what the peak will be. And if the object were in fact on an impact trajectory, the probability would eventually climb to 100%. However, the expectations can be guided by sampling the orbital uncertainty and see how the corresponding impact probability evolves with simulated future data (Fig. 8).

The collaboration between the Sentry, Aegis, and NEODyS teams proved beneficial. The differences between the systems allowed us to assess the robustness of the results, especially as the significance of the risk increased. The comparison of the results and discussion on the different assumptions provided an informal peer-review process that greatly increased the confidence of the impact monitoring calculations.

Over the years, hypothetical impact scenarios have been developed to conduct planetary defense exercises to test the readiness to respond to an asteroid impact threat.^22^ The discovery, orbit determination, and impact hazard assessment components of those exercise are generally assumed to work based on the fact that, unlike other aspects, those components are exercised on a daily basis. However, going through the process for 2024 YR_4_ proved valuable to maximize the elements of realism: real discovery circumstances, real observational data from observers with possible unexpected problems, actual difference in the results to be understood, etc. The experience proved satisfactory, with a stable orbital evolution and an impact probability evolution following theoretical expectations. Another aspect that was validated was that of using a space-based infrared telescope like JWST, not only to obtain physical properties, but also to extend the astrometric data arc beyond what ground-based assets achieved.

There were no radar detections of 2024 YR_4_. Goldstone could have observed the asteroid right after discovery, but at that time 2024 YR_4_ did not yet stand out as a highly significant impact risk. Had it not collapsed [34], the Arecibo radar observatory might have been able to detect 2024 YR_4_ until early January. To highlight the power of radar data, we simulated the contribution of radar observations taken within a week of discovery. Radar would have forced the Torino Scale to be 0 and the impact would have been ruled out shortly after discovery, instead of having to wait for two months of optical tracking.

If NASA’s NEO Surveyor [35], which is planned for launch no earlier than 2027, had been operational, it could have detected 2024 YR_4_ in October or November 2024. A similar conclusion holds for NEOMIR [36]; had it been operational in late 2024, NEOMIR could have discovered 2024 YR_4_ about a month before ATLAS. The corresponding arc extension would have helped decrease orbital uncertainties, especially during the earlier phases of the discovery apparition. Moreover, infrared measurements would have contributed to constraining the size of the asteroid.

Precovery data in 2016 and 2020 could have ruled out the possible impact on Earth much sooner and provide a better understanding of the possibility of a lunar impact. Unfortunately, attempts to detect 2024 YR_4_ in archival images proved unsuccessful [18]. In case the data arc extension had not ruled out the impact, a possible approach could have been that of a negative observation, i.e., showing that the asteroid is not where it would be if it were on an impact trajectory [37, 38]. This approach can be adopted for recovery efforts where the total ephemeris uncertainty is too large to cover, but the footprint of the impacting solutions is sufficiently small. The same approach can be used for precovery images. However, negative observations can be challenging because one needs to ensure that the image went sufficiently faint to detect the asteroid, that the asteroid is not overlapping with field stars, and that the impact footprint is correctly mapped into the plane-of-sky. In particular, the exact location and extent of the footprint can be affected by nongravitational perturbations such as solar radiation pressure [39] and the Yarkovsky effect [40]. Due to these challenges, negative observations need to follow a formal protocol that includes a peer review.^23^

Based on population models [41], objects of the size of 2024 YR_4_ (60 m [32]) collide with Earth every $\sim$5000 years, on average. Therefore, the background impact rate is $\sim 2 \times 10^{-4}$/yr. At its peak, 2024 YR_4_ held a 3% probability of impact within 8 years, which corresponds to a rate of $4 \times 10^{-3}$/yr, $\sim$20 times larger than the background. However, the Palermo Scale [12], which is precisely meant to quantify a specific impact risk relative to the background, reached a maximum of -0.17, indicating that the risk from 2024 YR_4_ was only 70% of that from the background. In contrast, with reference to a modern estimate of the background flux, the Palermo Scale for 2024 YR_4_ would have been $+1.3$. This discrepancy is due to the population model used in the original definition of the Palermo Scale, which has a steeper power law than more recent population models [42] and does not capture the dip between absolute magnitudes 20 and 28 relative to the power law [6]. Both limitations lead to a significant overestimate (by $\sim$ $30\times$ combined) of the number of objects with a size similar to 2024 YR_4_ and their impact flux.

This paper highlights the challenges of performing asteroid impact monitoring, especially for high-risk cases. The CNEOS, NEOCC, and NEODyS teams had to meet requirements in terms of transparency, responsiveness, and accuracy. As new observational data were reported on a nearly daily basis, we vetted each observation, contacted observers whenever needed to request additional information, assessed the sensitivity of the orbital solution to data weighting and rejections, tracked the evolution of the orbital predictions mapped at the time of the 2032 close approach, and compared the impact probability estimates. The success of the 2024 YR_4_ campaign rested on the high fidelity of the observational data, and the technical expertise and collaboration between the impact monitoring teams based on over 20 years of experience.

## Supplementary Information

Below is the link to the electronic supplementary material.

Supplementary file 1

## Data Availability

Data is provided within the manuscript or supplementary information files

## Footnotes

1 https://cneos.jpl.nasa.gov/pi/.

2 https://cneos.jpl.nasa.gov/sentry/.

3 https://neo.ssa.esa.int/risk-list.

4 https://newton.spacedys.com/neodys/.

5 https://cneos.jpl.nasa.gov/news/news146.html.

6 https://cneos.jpl.nasa.gov/news/news148.html.

7 https://neo.ssa.esa.int/documents/20126/419169/Newsletter+February+2025.pdf.

8 https://www.minorplanetcenter.net/mpec/K24/K24YE0.html.

9 https://www.minorplanetcenter.net/mpec/K25/K25L19.html.

10 https://www.minorplanetcenter.net/.

11 The observation files used by the three systems are available as supplementary material in the ADES format.

12 https://cneos.jpl.nasa.gov/sentry/details.html#?des=2024%20YR4.

13 https://neo.ssa.esa.int/search-for-asteroids?tab=possimp&des=2024YR4.

14 https://newton.spacedys.com/neodys/index.php?pc=1.1.2&n=2024YR4.

15 https://www.minorplanetcenter.net/mpec/K24/K24YI9.html.

16 The overall Aegis and NEODyS Torino Scale for 2024 YR_4_ did not drop to 0 because the second VI for 2032 retained a higher impact probability in this time interval.

17 https://github.com/IAU-ADES.

18 https://iawn.net/index.shtml.

19 Animations showing the evolution of the 2024 YR_4_ position uncertainty relative to the the Earth are available here (https://science.nasa.gov/blogs/planetary-defense/2025/02/24/latest-calculations-conclude-asteroid-2024-yr4-now-poses-no-significant-threat-to-earth-in-2032-and-beyond/) and here (https://www.esa.int/Space_Safety/Planetary_Defence/Asteroid_2024_YR4_no_longer_poses_significant_impact_risk).

20 https://iawn.net/documents/NOTIFICATIONS/2024-YR4_IAWN_Potential-Impact-Notification_20250129.pdf.

21 https://iawn.net/documents/NOTIFICATIONS/2024-YR4_IAWN_Final-Notification_20250224.pdf.

22 https://cneos.jpl.nasa.gov/pd/cs/.

23 https://minorplanetcenter.net/media/documentation/negative_observations/VI_Observations_Protocol_3.1.pdf.
